# Effect of the Addition of Graphene Flakes on the Physical and Biological Properties of Composite Paints

**DOI:** 10.3390/molecules28166173

**Published:** 2023-08-21

**Authors:** Natalia Bartczak, Jerzy Kowalczyk, Robert Tomala, Mariusz Stefanski, Damian Szymański, Maciej Ptak, Wiesław Stręk, Konrad Szustakiewicz, Tomasz Kurzynowski, Łukasz Szczepański, Adam Junka, Damian Gorczyca, Paweł Głuchowski

**Affiliations:** 1 Institute of Low Temperature and Structure Research, Polish Academy of Sciences, PL-50422 Wroclaw, Poland; jerzy.kowalczyk@pwr.edu.pl (J.K.); r.tomala@intibs.pl (R.T.); m.stefanski@intibs.pl (M.S.); d.szymanski@intibs.pl (D.S.); m.ptak@intibs.pl (M.P.); w.strek@intibs.pl (W.S.); 2 Faculty of Chemistry, Wroclaw University of Science and Technology, PL-50370 Wroclaw, Poland; konrad.szustakiewicz@pwr.edu.pl; 3 Faculty of Mechanical Engineering, Wroclaw University of Science and Technology, PL-50370 Wroclaw, Poland; tomasz.kurzynowski@pwr.edu.pl (T.K.); lukasz.szczepanski@pwr.edu.pl (Ł.S.); 4 Platform for Unique Models Application, Wroclaw Medical University, PL-50367 Wroclaw, Poland; adam.junka@umw.edu.pl; 5 Medical Department, Lazarski University, PL-02662 Warsaw, Poland; damian.gorczyca@lazarski.pl

**Keywords:** graphene flakes, composite paints, acrylic paint, varnish, enamel, micro-hardness, wettability, antimicrobial properties

## Abstract

In this study, graphene flakes were obtained using an electrolytic method and characterized using X-ray diffraction (XRD), Raman and FTIR spectroscopy, scanning and transmission electron microscopy (SEM/TEM). Graphene-based composites with varying concentrations of 0.5%, 1% and 3% by weight were prepared with acrylic paint, enamel and varnish matrices. The mechanical properties were evaluated using micro-hardness testing, while wettability and antimicrobial activity against three pathogens (*Staphylococcus aureus* 33591, *Pseudomonas aeruginosa* 15442, *Candida albicans* 10231) were also examined. The results indicate that the addition of graphene flakes significantly enhances both the mechanical and antimicrobial properties of the coatings.

## 1. Introduction

Graphene is a well-known, single-layer, two-dimensional material. Its Young’s modulus is 1 TPa [1], and its tensile strength is up to 130 GPa [2]. Graphene is one hundred times more durable than steel [3], and it is believed to be a nearly perfect conductor of heat and current. It also has high chemical resistance and thermal stability. Because of its favorable physical and chemical properties, it has been recognized as a very promising material, which can replace commonly used materials. It may occur in the form of pure graphene, graphene oxide (GO) or reduced graphene oxide (rGO) [4]. Graphene was synthesized from graphite for the first time through a mechanical exfoliation method using adhesive tape [5]. Nowadays, there are numerous methods of producing graphene from cheap graphite. For example, there are a few distinguishable chemical methods involving nitric or sulfuric acid, chemical vapor deposition (CVD), epitaxial growth, electrodeposition or electrolysis, which is examined herein. Even though most of these methods give satisfactory results in laboratory conditions, there is still a substantial demand for producing graphene on an industrial scale. Many attempts are also undertaken to produce multi-functional and price-attractive product from a cheap source, such as graphite [4].

Thanks to high electron mobility, thermal conductivity and biocompatibility, graphene structures have found application in many areas, such as electronics [6,7,8], sensors [9,10], solar panels [11,12] or drug delivery [13].

Due to its superior mechanical properties, graphene-based composites with various polymers or graphene-based paints and varnishes are widely studied [14,15,16,17]. Presently, a substantial pursuit is being noticed of multi-functional coatings marked as reliable and durable. The issue that scientists must face is the provision of protection against water, oxidation, corrosion or micro-organisms. There is also a high interest in the development and manufacture of self-healing, self-cleaning, anti-fouling surfaces [18,19,20]. Corrosion inhibitors have been well known so far, but there is a distinctive need to extend the knowledge about anti-corrosion graphene-based coatings. This unfamiliar field is leading scientists to extend their research, and as a result, the global market can count on the latest reports [21]. It has to be stressed that the addition of graphene may be the answer to improved surface functionality. Thus, to overcome metal corrosion problems—which can be caused by acid or wet conditions—fouling issues and to improve anti-bacterial properties, scientists have carried out studies with superhydrophobic (SHP) coating of nickel (Ni)-reduced graphene oxide (rGO)-myristic acid [21]. In many reviews, protective graphene films were applied on surfaces as an alloy, e.g., with aluminum, nickel, copper, etc. [22,23,24]. Although research showed a reduced rate of corrosion of metals in solutions (for example, sodium sulfate solution) and corrosion inhibition under biological action, in almost one hundred percent of the cases, the method has a few main drawbacks, e.g., graphite’s weak long-term protection of metal surfaces or a variety of stable structures of different metals [23]. Another method involved adding graphene or its derivative (graphene oxide (GO) or reduced graphene oxide (rGO)) as a filler into protective coatings [23]. Such an approach was proved to provide excellent protection on epoxy coatings, but on the other hand, there was a lack of compatibility between resin and graphene. Another problem pertained to graphene’s high surface energy, which led to agglomeration. To solve the issue, dispersed graphene composites were taken and finally applied on protective coatings [25,26]. Nowadays, graphene-based paintings and varnishes gain more popularity due to their comprehensive effects. They have been recognized as significant antioxidants, anti-scratch and anti-UVA ray remedies [27,28,29]. For this reason, they are believed to be the future of civil engineering, aviation, chemical and electrical industry. Graphene is expected to be used in many applications, starting from the most prosaic—such as anti-bacterial coatings; paints, which protect the walls against heat loss or absorbing solar energy; coatings with the ability to ban UV rays and, as a result, to decrease radiation through the windows; as support for non-sticky surfaces—and ending on the most future-proof, such as graphene-based coatings, which change walls into interactive screens, but this issue is still being tested. The success of graphene-based coats can be attributed to their flexibility, affordability, design and their potential to overcome the limitations of traditional coats. Modifications were also made in ancient architectural art. An attempt was made to improve the exhibit’s resistance to corrosion and UV light by testing FEVE (trifluoro vinyl chloride and vinyl ether copolymer) paint introduced by graphene oxide m-GO [29]. Most of the scientific reports are focused on its anti-corrosion properties. After using graphene and its derivatives as a barrier film directly applied on steel, scientists’ curiosity turned onto graphene-based composites—not only for industrial but also potential domestic use. So far, the differences between polymer and graphene-based coatings have been studied, and it turns out that the polymer has a much weaker adhesion to the surface; it interrupts the coat’s functionality (for instance, thermal and electrical conductivity), not to mention the rapid biodegradation. Graphene-based covers, in contrast to polymer ones, prevent microbially induced corrosion, which is caused by micro-organisms on many surfaces [30]. Thus, trials were performed in graphene-based composite coats’ development. Scholars had an idea of mixing functionalized graphene in resins or polymeric coats. In all cases quoted in this part, the coats gained high corrosion resistance and good functional properties. The first patents for graphene-based coatings have been established, which order graphene functionalization and use such organic compounds as urethane, epoxy, acrylic, alkyd resins. There have been cases of supplementing graphene oxide (GO) and reduced graphene oxide (rGO) to diluted acrylic resin, and as a result, an equal introduction of graphene flakes has been achieved using graphene powder [31]. Acrylic coats modified with functionalized graphene oxide (fGO) applied on steel favor conductivity and increased resistance in chemically aggressive conditions [32]. The addition of graphene nanoflakes to epoxy-based polyurethane coatings amends degradation resistance in UV radiation [27]. Due to electrical conductivity and lightning strike protection, graphene has been initiated as a nanofiller in water-based epoxy resin and applied on the most common aircraft alloy, improving barrier properties [33]. A suspension with reduced graphene oxide ensures mechanical stability, durability and superhydrophobicity, despite scratching [34]. There are examples of developing epoxy anti-corrosion coatings with graphene oxide and polyaniline GO/PANI. Such composite was distinguished with an excellent dispersion ability, thermal stability and anti-corrosion features [35]. To extend the view, graphene/Fe_3_O_4_ composite varnish with higher than ordinary paint corrosion resistance was invented, providing great coverage for ships or urban exteriors [15]. The variety of properties and not yet fully discovered potential of graphene-based composites have encouraged authors to broaden the knowledge about applications and possible use of this precious fabric in paintings and varnishes. The marketplace can be proud of the variety of paints—for instance, graphene-based lime paints—which, in addition to their excellent resistance and protective properties, also have carbon-dioxide adsorption ability [36].

For this reason, and to take advantage of graphite electrolysis’s simplicity and low costs, graphene flakes were examined herein. An attempt was made to prepare graphene-based paints and varnishes in order to study graphene’s mechanical and biological properties and to further evaluate its potential applications.

## 2. Results

### 2.1. Structure and Morphology of Graphene Flakes (XRD, Raman and FTIR Spectroscopy, SEM, TEM)

**Figure 1:**
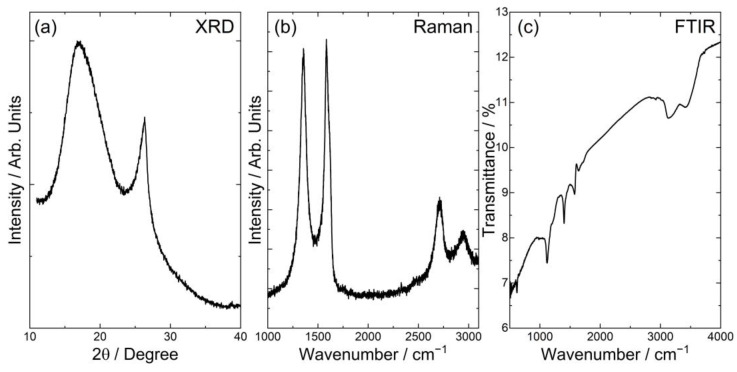
XRD pattern (**a**), Raman spectrum (**b**) and FTIR spectrum (**c**) of the graphene flakes.

The XRD pattern (Figure 1a) shows a broad peak between 10 and 30° and a narrower one at around 27°. The first is associated with the reflection of X-rays from the sp^2^ planes, while the second is observed due to the crystalline structure of graphite, which still exists in the sample. The broad band observed in the diffractogram indicates that the graphene observed here takes the form of reduced graphene oxide (rGO) [37]. Based on the registered position of the diffraction peak for graphene flakes and the Bragg equation, the inter-layer spacing was calculated. For the calculation, values of 0.154 nm (the wavelength of the X-ray beam) and 16.5° (2Θ degree) were utilized, resulting in a distance between the layers of approximately 5.37 Å. Utilizing the Scherrer equation [38], the average size of the flakes was estimated to be 1.55 nm. However, it should be noted that the Scherrer equation is not suitable for calculations involving 2D structures; it is intended for use with spherical particles below 100 nm. Therefore, it is important to acknowledge that these calculations are rather approximate. Nevertheless, considering the calculated value and the inter-layer distance determined, an estimation can be made that the graphene flakes consist of about three layers of graphene. This observation aligns with findings from the TEM images. The Raman spectrum (Figure 1b) shows three characteristic peaks: D (1350 cm^−1^), G (1580 cm^−1^) and 2D—also referred to as G’ (2700 cm^−1^) [39]—and an additional one at about 2950 cm^−1^, which is associated with the D + G mode. The 2D band intensity is lower than the G band, while the D band has a similar intensity as the G band. The ratio of the D band to the G band indicates the degree of deformation [40] and is equal to 0.95. The lower intensity and narrow character of the 2D band are evidence of a multi-layer graphene structure. It is known that the ratio I_2D_/I_G_ is dependent on the number of graphene layers [41]. As the 2D band is the characteristic peak of the graphene structure, the higher intensity of this peak compared to the G band indicates fewer layers in the structure. The ratio I_2D_/I_G_ ∼ 2–3 is observed for mono-layer graphene, 2 > I_2D_/I_G_ > 1 for bi-layer graphene and I_2D_/I_G_ below 1 for multi-layer one. The I_2D_/I_G_ ratio for rGO in the studied material is 0.44, which confirms the multi-layer character of the prepared flakes. The FTIR spectrum (Figure 1c) is quite difficult to analyze, as it was measured in the transmission mode using a KBr pellet. Due to the difficulty in grinding graphene flakes, the IR signal is weak, and the observed background is high. However, the spectrum can still provide interesting information. Broad bands at about 3141 cm^−1^ and 3433 cm^−1^ are evidence of the presence of O-H hydroxyl groups, which can arise from water molecules attached to the graphene surface but also from the high hygroscopicity of KBr. The absorbed radiation displayed as a spectrum makes it possible to identify functional groups in the graphene sample. In the range of 1130 cm^−1^ and 1577 cm^−1^, stretching vibrations of C-O and C=C, respectively.

**Figure 2:**
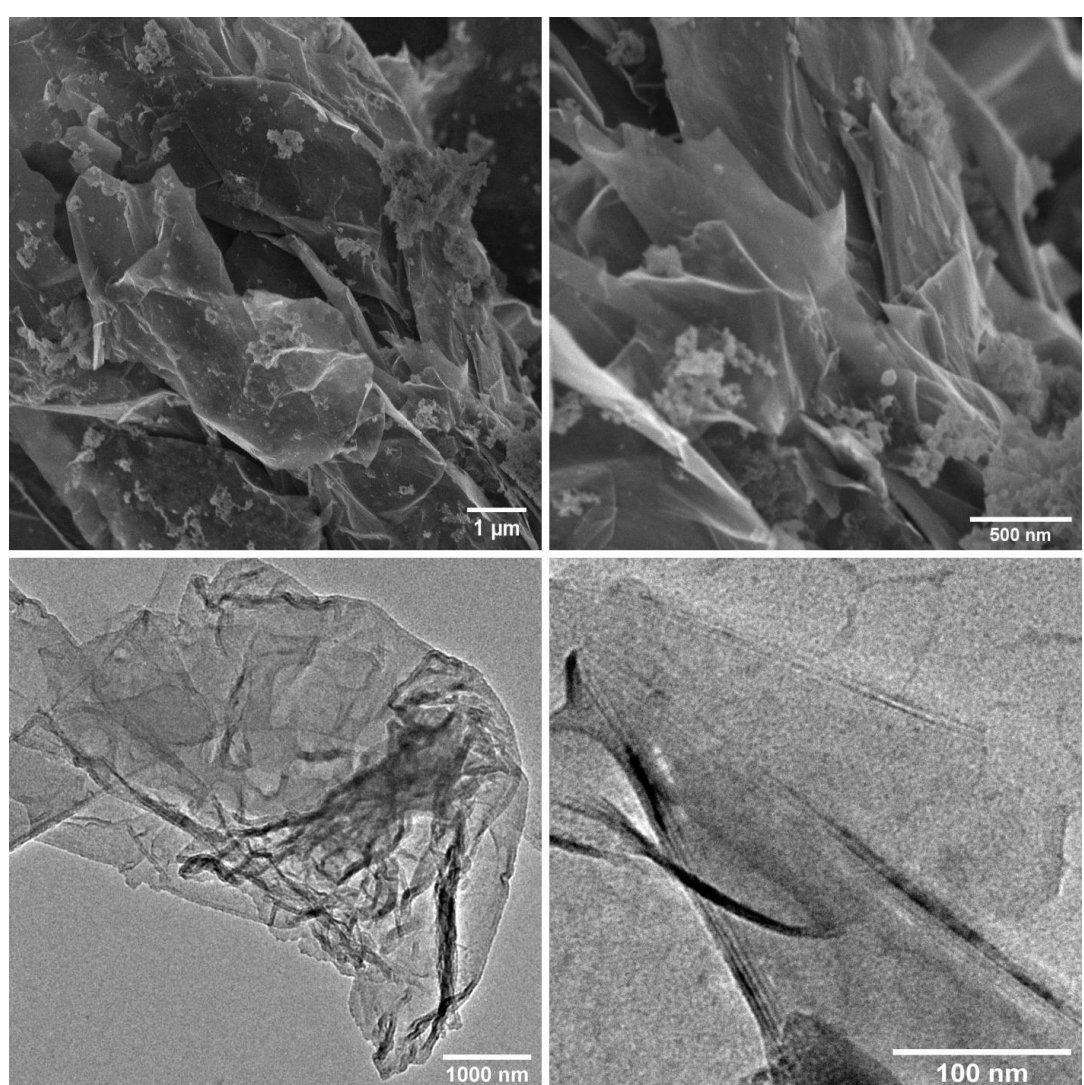
SEM (**top**) and TEM (**bottom**) images of graphene flakes used for composites’ preparation.

The SEM images of graphene flakes used as a filler in the composites are shown in Figure 2 (top). The images reveal a multi-layered structure of the graphene flakes. It should be noted that the flakes have a relatively high size distribution, which can affect their dispersibility in the composite paints. The smaller flakes, due to their better colloidal stability, may be more easily incorporated into the composite materials. On the other hand, larger flakes have a higher impact on the strengthening of mechanical properties, and their edges protruding beyond the surface of the polymer matrix can act as blades, which damage the bacterial cell membrane, leading to their apoptosis. The TEM images (Figure 2 (bottom)) show that the size of the flakes is in the ten square micrometers range. The obtained flakes have an uneven, irregular shape. The more transparent areas indicate a single graphene layer, while the darker ones show a stack of a few layers [42]. The wrinkled structure on the edges of flakes is the result of the sonification process, which removed the intercalated oxygen and sulfur groups [43]. On the edge of the enlarged fragment of the TEM image (Figure 2 (bottom right)), the fringes corresponding to successive layers of graphene can be seen, indicating that the flakes may consist of several layers (from two to five).

### 2.2. Physical Properties of the Composites

#### 2.2.1. Morphology of Composite Paints’ Surface

**Figure 3:**
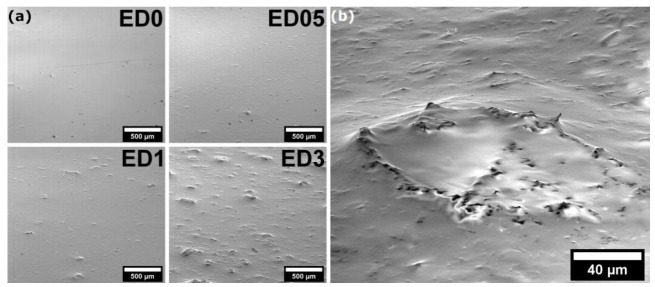
SEM images of the surface of enamel paint and composites (**a**) and magnification of the graphene flakes’ aggregates with visible “blades” (**b**).

For the enamel composite coatings, SEM images were taken to show how the graphene flakes are arranged on the surface (Figure 3). It can be seen that flat “islands” appear on the smooth surface of the enamel after adding graphene flakes. For the smallest concentration of flakes, it can be observed that they are mostly arranged in a plane-parallel manner, creating small spots on the surface of the composite. At a higher concentration of graphene flakes, aggregates appear, creating large irregularities, with flakes protruding on their surface, in many cases set perpendicularly to the surface of the coating. At higher magnification (Figure 3b), it can be seen that some of the flakes protruding above the surface of the layer create a kind of “blade”, which has a significant impact on the physical and biological properties of the obtained coatings.

#### 2.2.2. Micro-Hardness and Elasticity

**Figure 4:**
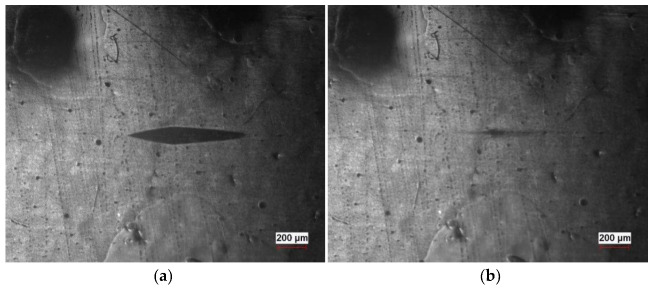
The micro-indentations on a ED05 composite surface: immediately after measurement (**a**) and 10 s later (**b**).

Knoop micro-hardness measurements were conducted for each composite, and the recorded results were limited to the enamel samples. The ED3 sample displayed the highest micro-hardness, with comparable values obtained for the reference sample (Table 1). Micro-hardness measurements indicated that the addition of graphene at low concentrations did not significantly enhance the coating hardness, although an increasing trend was observed with higher graphene concentrations. It is possible that enamel samples with graphene concentrations exceeding 3 weight% may exhibit higher micro-hardness. The results were notably influenced by the increase in layer flexibility after the addition of graphene, as depicted in Figure 4. Due to the substantial surface flexibility of acrylic and varnish coats, measurements could not be performed. In the case of varnish samples, the imprint of the indentation rapidly disappeared, resulting in poorly visible indentation edges, rendering accurate micro-hardness interpretation and determination of the layer hardness impossible. Similarly, in the case of acrylic graphene-based paints, the indentation made by the diamond indenter disappeared immediately before the indenter returned, leaving no imprint on the surface.

#### 2.2.3. Wettability

**Figure 5:**
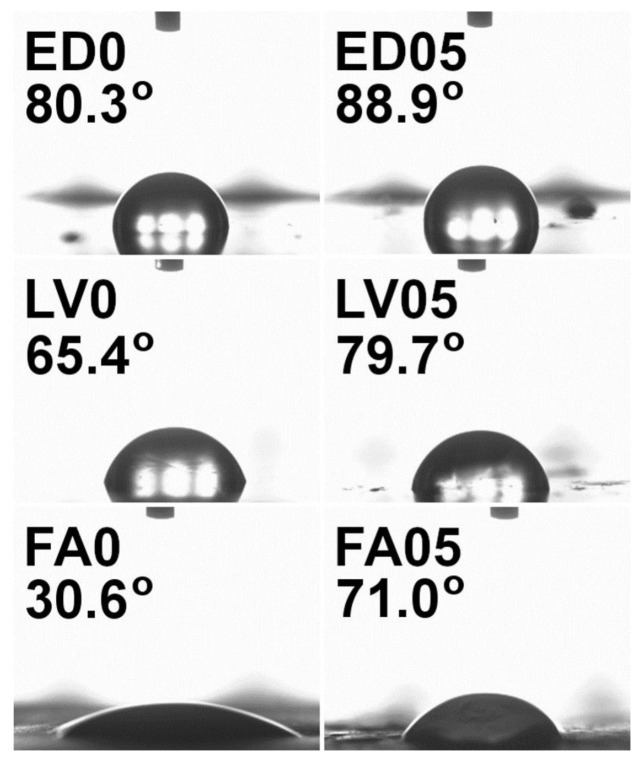
Water contact angle pictures of reference surface (XX0) and composite paint with 0.5 weight% graphene (XX05). ED—enamel, LV—varnish, FA—acrylic paint.

The wettability tests conducted on graphene-based varnish, enamel and acrylic composites revealed contact angles of less than 90°. Water droplets were observed to remain on the surfaces of enamel and varnish, while on the acrylic paint surface, the droplets tended to flow, and in some areas, they were even absorbed by the coating. Multiple measurements were taken at different locations to obtain an average result. However, due to the weak dispersion of graphene flakes within the composite volume and partial aggregation of flakes in certain areas (as seen in the SEM images), the measurements were subject to a significant measurement error. This was particularly evident in composites with higher graphene content (1% and 3% by weight), where the wettability of the composite was sometimes lower than that of the pure paint (Table 2). In contrast, composites with lower graphene content (resulting in better dispersion and reduced aggregation) exhibited a notable increase in the wettability angle for each paint, indicating an increase in the hydrophobicity of the layers (Figure 5).

#### 2.2.4. Biological Properties of the Composites

**Figure 6:**
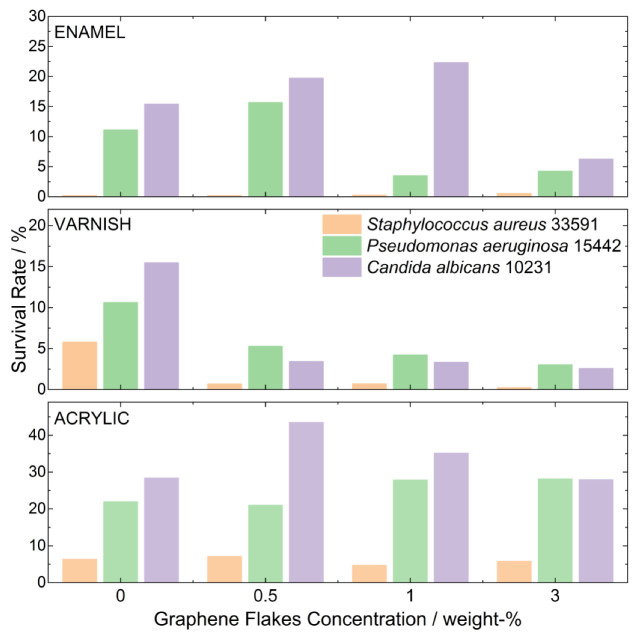
Survival rates for three pathogens on the different paint composites prepared with various concentrations of graphene flakes.

Antimicrobial tests performed on the composites were carried out to check their activity against three pathogens: *Staphylococcus aureus* (Gram-positive bacteria), *Pseudomonas aeruginosa* (Gram-negative bacteria) and *Candida albicans* (fungi). Octenidine dihydrochloride was used as a control antimicrobial agent with proven activity against these pathogens, where the reduction in living cells was close to 100%, and it was set as a reference for the activity of composites. The lowest antimicrobial activity was revealed for the acrylic composite paint. Only in a few cases was the reduction in bacteria higher in composites compared to pure paint. There was also no clear tendency in the pathogen survival rate with the concentration of graphene flakes in the composites (Table 3). In the case of enamel composites, the antimicrobial activity was higher, and it can be observed that by selecting the appropriate concentration of graphene flakes, it was possible to obtain active coatings against all types of pathogens tested. The highest antimicrobial activity was exhibited in varnish composites. The LV3 composite reduced the pathogens on the surface by over twenty times the amount of Gram-positive bacteria, three times the amount of Gram-negative bacteria and five times the amount of fungal cells. It should be noted here that the composite displayed very high activity against the bacterial and fungal pathogens tested, which are considered the leading etiological factors of a broad spectrum of community and hospital infections. In the case of varnishes, even the lowest concentration of graphene flakes improved the antimicrobial properties of the composites (Figure 6).

## 3. Discussion

The results of measurements of composites based on graphene flakes clearly show that their physical properties can be effectively modified by addition of graphene flakes. The micro-hardness tests show that acrylic graphene-based paints exhibit the highest flexibility among the examined paints. These observations are in line with the results obtained by Chang-Tsan Lu et al. [44], as they showed that the increase in composite modulus scales linearly with the flake size (circular flake radius). Similar results were shown for the PVDF-HFP copolymer, where increasing the concentration of the larger size graphene flakes increased Young’s modulus and the tensile strength of the composite [45]. The results provide valuable insights into the potential future applications of graphene-based paints, particularly in self-healing coating formulations, owing to their high elasticity and low hardness at low concentrations. Additionally, the wettability tests of composites showed a positive effect of the addition of graphene flakes. As shown in the SEM images, for a low concentration of graphene flakes, they are mostly arranged flat on the surface, and, thanks to their properties, they increase the hydrophobicity of the entire surface. At higher concentrations of the flakes, they can be observed on the surface positioned with the edge up, which, when in contact with water, can lead to the introduction of stresses into the water drop and a decrease in its surface tension. Decreased wettability of the composites at low graphene flake concentrations has been reported previously in studies involving epoxy resin, where the addition of 0.5% and 1% graphene resulted in an increase in water contact angles by 6.2° and 16.1°, respectively [46]. The decrease in wettability of the composite layers containing graphene results from the hydrophobic nature of graphene itself. Chih-Jen Shih et al. [47] showed that the wetting behavior of graphene-coated surfaces is dependent on the number of graphene layers as a result of its surface tension and the strength of the inter-layer van der Waals interactions. As a consequence, the higher contact angle on the mono-layer results from its lower surface tension. They also showed that the addition of one or two graphitic layers to the mono-layer increases the total van der Waals interaction potential and thus reduces the contact angle. Taking into account the above considerations and the results obtained for the composites, it can be assumed that, in the case of a lower concentration of graphene flakes in the composite, they are better dispersed with individual flakes in the paint volume, while higher concentrations cause the aggregation of flakes and the formation of multi-layer structures in the volume of the composite. Therefore, the highest contact angles are observed for composites with the lowest concentration of graphene flakes.

The wettability of the composite surface also has an enormous impact on the antimicrobial properties of the obtained materials. The preceding section demonstrates that the wettability of the acrylic paint composite is notably elevated (rendering the composite more hydrophilic) in contrast to alternative paints. Given that the contact angle exerts a substantial influence on the micro-biological activity of the composite [48] (increased hydrophobicity could potentially constrain the accessibility of graphene “blades” to the cytoplasmic membrane of bacteria), the micro-biological activity of acrylic coatings experiences a considerable reduction. It should be noted that the effect of antimicrobial activity of an active compound in any composite is influenced not only by microbial sensitivity to the compound but also by the compound’s distribution on/in the material, the compound’s mechanism of action and microbial inter- and intra-species differences manifesting in the broad spectrum of sensitivity/tolerance to a given antimicrobial. All these factors contribute collectively to the overall micro-biological result. The antimicrobial activity of graphene-based composites is based on several mechanisms. Numerous studies on carbon-based nanomaterials have claimed that the formation of reactive oxygen species (ROS) is the primary mechanism of antimicrobial activity. The ROS species produced from graphene and its derivatives are considered to be generated through interaction with oxygen or other electron transport chain carriers (e.g., NADH, NADPH or FADH2) [49]. Another proposed mechanism is bacterial membrane damage by extraction of phospholipids caused by van der Waals interactions between the graphene sheets and the hydrophobic moieties of the membrane phospholipid bi-layer. The exposure of these lateral edges to bacterial membranes has been found to cause movement of phospholipids along the membrane, resulting in lipid extraction [50]. Another simpler approach for explaining the antimicrobial activity of graphene flakes is their morphology. Recent studies show that the action of sharp edges—also called nano-knives, cutters or blades—is one of the most crucial factors affecting the antimicrobial properties of graphene materials [51]. Most of the carbon derivatives were tested in the form of powder exposed to interaction with bacteria. In the presented case, the graphene flakes are caged in the composite, and therefore, not all mechanisms can be applied here. Based on the SEM images of the surface of composite paints, higher concentrations of graphene flakes lead to more “blades” appearing, and the most likely mechanism in the case of paint composites, which leads to apoptosis of bacterial or fungal cells, is the physical contact of the edges of graphene flakes with the cell walls and their physical damage. This conclusion is supported by the fact that, in most cases, the highest activity is shown by composites with the highest concentration of flakes, which means that they exhibit the most “blades” on their surface. The works of other research groups indicated high antimicrobial activity of composites based on graphene/GO and its derivatives, although in most cases, the coatings used systems where graphene was conjugated with silver nanoparticles, which also exhibit anti-bacterial properties (Table 4). Anti-bacterial tests for graphene itself were mainly carried out for powder materials [52,53,54] or graphene/GO layers applied directly to another structure (e.g., titanium [55]), but in this case, both the type of layer, its application and interactions with micro-organisms are of a completely different nature.

The physical and biological properties of acrylic paint, enamel and varnish composites after the addition of graphene flakes were evaluated in this study. The graphene flakes used in this study were characterized as being of good quality with a large size, which posed challenges in achieving well-homogenized materials. The varnish and enamel composites showed notable hydrophobic properties, while all graphene-based paints exhibited low micro-hardness. However, the rapid disappearance of indentation observed during micro-hardness tests suggested a high potential for developing graphene-based coatings with self-healing properties. Additionally, antimicrobial tests showed a significant pathogen reduction on the surface of the paints, even for very low concentrations of the graphene flakes. The physical and biological measurements shed new light on the development of graphene-based composite paints. To fully realize their potential, further solutions need to be implemented to make them fully functional and suitable for commercial use.

## 4. Materials and Methods

The graphite foil (GFC99M1—0.1 cm thick, Sinograf Toruń S.A., Toruń, Poland) was cut and prepared for stripes with dimensions of 20 × 100 mm. The 0.1 M aqueous solution of ammonium sulfate (purity > 97%, Chempur, Piekary Slaskie, Poland) was used as a reaction medium in electrolysis. For the composite paints, commercial products were used: acrylic paint (Snieżka, Śnieżka Trade of Colours Ltd, Warsaw, Poland), varnish (Vidaron, Śnieżka Trade of Colours Ltd, Warsaw, Poland), enamel (Dekoral, PPG Deco Polska Sp. z o.o., Wroclaw, Poland). The reference strains of methicillin-resistant *Staphylococcus aureus* 33591, *Pseudomonas aeruginosa* 15442, *Candida albicans* 10231 (American Type and Culture Collection) were incubated for 24 h at 37 °C in Tryptic Soy Broth (TSB).

The graphene nanoflakes were prepared using the electrochemical method previously described by us in detail in Ref [61]. The two graphite foil strips (cathodes) were placed in a beaker with an aqueous solution of 0.1 molar ammonium sulfate, and one anode (graphite block) was placed between them. The beaker was then placed in an ice bath to set the electrolysis temperature to about 5 °C. The anodes were activated for 10 min at 2.5 V. Afterward, the voltage and current were increased to 10 V and 2.5 A, respectively, and the electrolysis process was carried out for 1 h. At the end of electrolysis, the solution of graphene flakes in aqueous ammonium sulfate was filtered using a soft filter and washed with distilled water five times. The resulting material was suspended in water and sonicated using a high-power homogenizer (Tefic Biotech Co., Limited, Xi’an, China, Laboratory Ultrasonoficator TF-900N) for 15 min at 700 W and then left for a minimum of three days. After 72 h, the suspension was separated from the decant, transferred to a new container and frozen. The frozen sample was then freeze-dried (Tefic Biotech Co., Limited, Freeze-dryer TF-10A) for seven days at a temperature of −52 °C and a pressure of 10^−3^ mbar.

The structure, morphology and quality of graphene flakes were examined using several techniques. X-ray diffraction measurements were carried out using Cu Kα monochromatic radiation (λ = 1.5406 Å) on an X’Pert PRO, PANalytical (Bruker, Karlsruhe, Germany) by scanning the diffraction angles (2θ) between 10° and 40° at room temperature in the Bragg–Brentano geometry. The Raman spectra were measured using a Renishaw InVia Raman spectrometer equipped with an optical microscope (DM 2500 Leica, 20× objective), a thermoelectrically cooled charge-coupled device (CCD) as a detector and an argon laser operating at 514 nm as an excitation source. The spectrum was measured in the 1000–3200 cm^−1^ range with a resolution of 2 cm^−1^. The IR spectra in the mid-IR range of 4000–400 cm^−1^ were measured using a Nicolet iS50 infrared spectrometer with the KBr pellet method. Scanning and transmission electron microscopies (FE-SEM—FEI NovaNano SEM 230 (FEI Company is part of Thermo Fisher Scientific, Waltham, MA, USA); TEM-Philips CM-20 SuperTwin, Eindhoven, The Netherlands) were used to study the morphology of graphene flakes. Samples for SEM and TEM measurements were prepared by dispersing a small amount of specimens in methanol and putting a droplet of the suspension on a carbon stub or copper microscope grid covered by carbon, respectively.

Dry graphene flakes were added into acrylic paint (FA), enamel (ED) and varnish (LV) to obtain composites with 0.5 (FA05, ED05, LV05), 1 (FA1, ED1, LV1) and 3 weight % (FA3, ED3, LV3) graphene concentrations. For this purpose, 0.25, 0.5 and 1.5 g of graphene flakes were added to paints (up to 50 g) for each concentration, respectively. The paint with the filler was mixed in a laboratory stirrer for 20 min, starting with the lowest rotations and finishing with the maximum 830 RPM. Afterward, metal plates with dimensions of 30 × 50 × 2 mm were covered with graphene-based composites for future studies.

The morphology of the enamel composite surface was investigated by FE-SEM. In the first step, the samples were placed on the stub in order to eliminate charging and drift problems. Afterward, the samples were put under an electron beam and analyzed by using a secondary electron (SE) for the stub tilted at an angle of 70 degrees in order to show more detailed features of the samples. The SEM images were recorded at 5.0 kV in a beam deceleration mode, which improves imaging parameters, such as resolution and contrast.

The micro-hardness measurements of dried samples were performed using the Knoop method, according to the ASTM E92 standard, using the ZHVµ-A (Zwick-Roell, Leominster, UK) hardness tester. The tests were conducted after establishing the optimal load, which allowed the measurement of the diagonals of the resulting imprint. For the indentations, 50 g load was applied for 10 s. Five measurements were conducted on the top surface of the samples. The wettability/water contact angle of the obtained composites was measured using an PGX goniometer (Thwing-Albert Europe, Deerlijk, Belgium) with integrated software, using the sitting drop method in the static mode, where the automatically released drop of deionized water with ~5 µL was placed on the surface of the measured sample.

To conduct antimicrobial studies, the tested paints were evenly spread into the wells of a 6-well plate (Biofil, Indore, India) using a micro-biological spatula under aseptic conditions in a laminar chamber. The plate was then incubated at room temperature for 24 h to dry the paint. The reference strains of methicillin-resistant *Staphylococcus aureus* 33591, *Pseudomonas aeruginosa* 15442 and *Candida albicans* 10231 (American Type and Culture Collection, Manassas, VA, USA) were incubated for 24 h at 37 °C in Tryptic Soy Broth (TSB, Biomaxima, Lublin, Poland). A suspension of the test strains was prepared in saline with a density of 0.5 McFarland (1.5 × 10^8^ CFU/mL) and diluted a thousand times in the culture medium. Next, 1 mL of the suspension was added to the wells of the 6-well plate containing the test paints and incubated for 24 h at 37 °C under static conditions. To control for microbial growth, the suspension was also added to wells devoid of test substances in 6 replicates. After incubation, the medium was gently removed from the wells, and 1 mL of a 0.1% (*w*/*v*) solution of 3,2,5-triphenyltetrazolium chloride (TTC, Fluka, Buchs, Switzerland) in TSB was added to the wells. The system was then incubated again at 21 °C for 4 h. Next, the medium was removed from the plate wells, and 1 mL of a 9:1 mixture of methanol and acetic acid was added. The plate was incubated at room temperature for 30 min with shaking (350 RPM). From each well, 100 µL of the solution was withdrawn three times and transferred to 3 wells of a 96-well plate. The absorbance of the solutions was measured spectrophotometrically at a wavelength of 490 nm, and the absorbance of each sample was determined by calculating the average of 3 measurements.

## Figures and Tables

**Table 1:** Micro-hardness of the composites.

Sample	Micro-Hardness	Standard Deviation
	HK	
ED0	2.8	0.4
ED05	1.0	0.1
ED1	1.2	0.4
ED3	2.4	0.49

**Table 2:** Contact angle measured for composite paints.

Sample	Contact Angle	Standard Deviation
	°	
ED0	80.3	3.1
ED05	88.9	0.8
ED1	76.0	5.2
ED3	71.4	3.5
LV0	65.4	2.0
LV05	79.7	3.4
LV1	71.6	3.4
LV3	61.1	4.0
FA0	30.6	8.1
FA05	71.0	2.2
FA1	33.7	9.1
FA3	44.8	14.4

**Table 3:** Antimicrobial properties of composite paints.

Sample	*S. aureus*		*P. aeruginosa*		*C. albicans*	
	Average Absorbance	Survival Rate	Average Absorbance	Survival Rate	Average Absorbance	Survival Rate
	Arb. Units	%	Arb. Units	%	Arb. Units	%
ED0	0.0544	0.22	0.2440	11.14	0.3730	15.43
ED05	0.0541	**0.20**	0.3438	15.70	0.4773	19.74
ED1	0.0557	0.27	0.0768	**3.51**	0.5397	22.32
ED3	0.0623	0.56	0.0936	4.27	0.1520	**6.29**
LV0	0.1849	5.81	0.2330	10.64	0.3743	15.48
LV05	0.0657	0.70	0.1160	5.30	0.0833	3.45
LV1	0.0660	0.71	0.0927	4.23	0.0811	3.36
LV3	0.0549	**0.24**	0.0668	**3.05**	0.0627	**2.59**
FA0	0.1984	6.39	0.4810	21.96	0.6870	28.41
FA05	0.2159	7.14	0.4607	**21.03**	1.0523	43.52
FA1	0.1596	**4.72**	0.6103	27.87	0.8500	35.15
FA3	0.1860	5.85	0.6170	28.17	0.6760	**27.96**

The highest antimicrobial activity for each composite and type of microbes is bolded.

**Table 4:** Comparison of antimicrobial activity of various graphene-based composites.

Composite	Type of Micro-Organism	Survival Rate/Time	Proposed Mechanism	Ref.
Acrylic paint + Ag + rGO	*E. coli* *S. aureus*	~5%/24 h	Interaction between Ag NPs and thiol groups in the enzyme of the bacterial cell membrane	[14]
Epoxy resin + rGO	*E. coli*	~35%/24 h	Oxidative stresses	[56]
PVA + Ag@GO	*E. coli*	0%/12 h	Interaction between Ag NPs and thiol groups in the enzyme of the bacterial cell membrane	[57]
PMMA + graphene	*S. aureus*	85%/7 h	Extraction of phospholipids from bacteria cell membranes	[58]
Acrylic paint + Ag@GO	*P. aeruginosa* *S. aureus*	0%/3 h	Interaction between the cell membrane and the electron-rich surface of the GO sheets	[59]
Epoxy Ag-rGO- curcumine	*S. aureus* *C. albicans*	0%/12 h	Adherence of microbes to the nanocomposite surface causes microbial cell lysis	[60]
Acrylic paint + graphene	*S. aureus* *P. aeruginosa* *C. albicans*	4.7%/24 h 21%/24 h 28%/24 h	Mechanical (direct contact of sharp edge of graphene flake with bacteria/fungi cell)	This work
Varnish + graphene	*S. aureus* *P. aeruginosa* *C. albicans*	0.24%/24 h 3%/24 h 2.6%/24 h	Mechanical (direct contact of sharp edge of graphene flake with bacteria/fungi cell)	This work
Enamel + graphene	*S. aureus* *P. aeruginosa* *C. albicans*	0.2%/24 h 3.5%/24 h 6.3%/24 h	Mechanical (direct contact of sharp edge of graphene flake with bacteria/fungi cell)	This work

## Data Availability

The data presented in this study are available on request from the corresponding author.

## Sample Availability

Not available.
